# Automated urine analyzers: a comparative study of Atellica UAS 800 and UAS 60 with risk analysis

**DOI:** 10.11613/BM.2025.010707

**Published:** 2025-02-15

**Authors:** Anita Radman, Adriana Unić, Marijana Miler, Lara Milevoj Kopčinović, Alen Vrtarić, Marija Božović, Nora Nikolac Gabaj

**Affiliations:** 1 Department of Clinical Chemistry, Sestre milosrdnice University Hospital Center, Zagreb, Croatia; 2 School of Medicine, Catholic University of Croatia, Zagreb, Croatia; 3 Faculty of Pharmacy and Biochemistry, University of Zagreb, Zagreb, Croatia

**Keywords:** urinalysis, automation, risk analysis, UAS 800, UAS 60

## Abstract

**Introduction:**

This study compared analytical and technical performance of Atellica UAS 800 and UAS 60 and assessed potential patient risks if results were not reviewed by laboratory personnel.

**Materials and methods:**

The study included 463 urine samples collected from February to March 2024, analyzed on both analyzers within 2 hours by two laboratory operators. Results from the UAS 800, recorded after operator review, were considered as the reference and compared to UAS 60 results obtained before and after review. Data were evaluated using weighted kappa (kappa ≥ 0.6 considered acceptable). Technical comparison was based on operator assessment. For risk analysis 23 errors and four severity levels were defined.

**Results:**

After automatic image evaluation strong agreement was observed for calcium oxalate and yeasts (kappa: 0.83, 0.94), moderate agreement for red and white blood cells and epithelial cells (kappa: 0.75, 0.78, 0.75), weak agreement for bacteria, mucus and non-squamous epithelial cells (kappa: 0.57, 0.59, 0.40), and poorest agreement for hyaline and pathological casts and total crystals (kappa: 0.23, 0.07, 0.36). After review, kappa was acceptable for all parameters. Risk analysis identified 15 errors, with unrecognized total crystals and mucus being the most frequent (30.0%, 17.1%). Three errors were classified as intermediate risk (missing to report total crystal +1, mucus +1 and pathological casts ≥ +1), with none in high risk area. UAS 800 offers higher throughput and automatic sample aspiration, while UAS 60 uses manual aspiration.

**Conclusions:**

Atellica UAS 60 provides results comparable to UAS 800, quality of reported results remaining uncompromised even without operator review. It is suitable for low- to mid-volume laboratories and can serve as a backup in larger laboratories.

## Graphical abstract

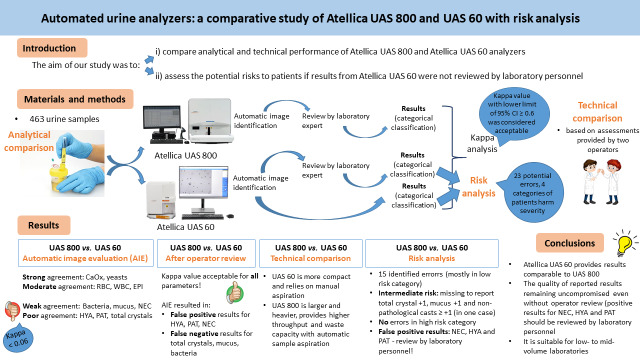

## Highlights

• Atellica UAS 60 provides results comparable to Atellica UAS 800

• Risk analysis identified 15 errors, mostly in the low-risk category, with unrecognized total crystals and mucus in category +1 being the most frequent, and no errors classified as high risk

• Positive results for non-squamous epithelial cells, hyaline and pathological cast after automated image evaluation should be reviewed by laboratory personnel

## Introduction

Urinalysis is one of the most commonly performed tests in clinical laboratories. It is almost essential in diagnosis, treatment and monitoring of various urinary tract disorders and kidney diseases, but also plays an important role in hepatic, metabolic and other systemic diseases (*1*-*3*). Urinalysis consists of two main steps: i) physicochemical testing, which includes urine visual examination and chemical testing using reagent strips, and ii) microscopic urine sediment analysis. Automation of urinalysis has been a key step in the standardization and improvement of the accuracy and reliability of urine test results, as well as in reduction of the analysis turnaround time, especially in high throughput laboratories (*4*-*6*). In recent years, automated urine analyzers have become widespread and almost inevitable in routine laboratory practice. They are typically composed of two integrated units: one for physicochemical testing and one for urine sediment analysis. Although several manufacturers have developed automated urine sediment analyzers, there are two main types based on their underlying principles: digital image-based systems and flow cytometry-based systems (*7*, *8*). These systems differ in several aspects, with one of the most significant being how results are displayed. Flow cytometry-based systems present results as scattergrams or isolated images of elements, which differ from microscopy and require substantial operator training. In contrast, digital image-based systems produce images closely resembling those seen in microscopy (*9*). In our laboratory, Atellica UAS 800 (Siemens Healthineers, Erlangen, Germany) is routinely used as a completely automated urine sediment analyzer. Recently, a new automated urine sediment analyzer, Atellica UAS 60, has been developed by the same manufacturer as a counterpart to the UAS 800. Both analyzers use the same digital imaging technology. In brief, 175 µL of the urine sample is pipetted into a cuvette and then centrifuged for 10 sec at 260xg to create a particle monolayer at the bottom of the cuvette. A built-in digital camera takes 15 digital images that closely replicates manual microscopy. Images are then evaluated with Auto Image Evaluation Module technology that digitally marks urine sediment particles (*10*, *11*). Currently, there are no studies on Atellica UAS 60 specifications or its comparison to UAS 800 analyzer. The aims of our study were to: i) compare the analytical and technical performance of Atellica UAS 800 and Atellica UAS 60 analyzers and ii) assess the potential risks to patients if results from Atellica UAS 60 were not reviewed by laboratory personnel, in accordance with the requirements of ISO 15189:2022 (*12*).

## Materials and methods

Before starting the study, we assessed the precision of the UAS 60 analyzer according to CLSI EP15-A3 using quality control materials KOVA Liqua-Triol Level I (Abnormal) i Level II (Normal) (Kova International, Garden Grove, USA, lot No: K306712) for RBC and WBC (*13*). Control materials were analyzed five times *per* day over five consecutive days. Both between-run and within-run precision were calculated. The acceptable criteria were set as perfect agreement with the manufacturer’s declaration for all measurements and precision thresholds of 15% for RBC and 13% for WBC.

### Study design

A total of 500 consecutive urine samples submitted for routine urinalysis between February 14 and March 7, 2024, from in- and out-patients at the Department of Clinical Chemistry, Sestre Milosrdnice University Hospital Center, Zagreb, Croatia, were included in the study. The samples were collected by clean catch and transferred to 10 mL VACUETTE Z Urine urine tubes with no additive (Greiner Bio-One, Kremsmünster, Austria). According to our current laboratory practice, each sample was first analyzed using Atellica UAS 800 (integrated with Clinitek Novus chemistry analyzer), followed by analysis on Atellica UAS 60 urine sediment analyzer (both Siemens Healthineers, Erlangen, Germany). Samples with insufficient volume for analysis on both analyzers, samples collected by catheters, and samples with more than two hours between collection and analysis on UAS 800 were excluded from the study. Additionally, 37 samples were excluded due to errors in automatic image evaluation on the UAS 60. Therefore, the total number of samples included in the analysis was 463. The Ethical Committee of the Sestre milosrdnice University Hospital Center approved this research (approval number: 003-06/24-03/026).

### Sample analysis

Maximum time from collection to analysis on UAS 800 was two hours, while maximum time from analysis on UAS 800 to analysis on UAS 60 was one hour. Before analysis, each sample was well mixed by 10-20 rotations by hand. Analysis on UAS 800 was performed from the primary sample tube, while for analysis on UAS 60, according to the manufacturer’s instructions, 175 µL of native urine was pipetted into a cuvette after routine urinalysis was completed and the sample had been mixed. All samples were evaluated by two skilled laboratory operators with over 20 years of experience in urine sediment analysis. After automatic image evaluation, laboratory operators reviewed each result and either confirmed or reclassified any misclassified urine sediment particles.

### Analytical comparison of Atellica UAS 800 and UAS 60 analyzers

The results (categorical classification) obtained from the Atellica UAS 800 after operator review were considered as reference. Results obtained from the UAS 60 analyzer were collected both before (after the first sample pass, automatic image evaluation) and after operator review and compared to the reference. The analyzed parameters included: red blood cells (RBC), white blood cells (WBC), WBC clumps, squamous epithelial cells (EPI), non-squamous epithelial cells (NEC), hyaline casts, pathological (non-hyaline) casts, bacteria, total crystals, crystals - calcium oxalate (CaOx), mucus, yeasts, and sperm. The reporting categories declared by the manufacturer for urine sediment results used to compare Atellica UAS 800 and UAS 60 are presented in Table 1.

**Table 1:** Reporting categories of urine sediment results used to compare Atellica UAS 800 and UAS 60

**Urine sediment parameter (p/µL)**	**Neg**	**+1**	**+2**	**+3**	**+4**
RBC	< 1.1	1.1-9.1	9.2-34.1	34.2-45.5	> 45.5
WBC	< 2.1	2.1-11.4	11.5-45.5	45.6-90.9	> 90.9
WBC clumps	< 2.1	> 2.1	-	-	-
EPI	< 1.1	1.1-5.7	5.8-17.1	17.2-27.3	> 27.3
NEC	< 0.1	0.1-0.9	1.0-1.4	1.5-1.8	> 1.8
Hyaline casts	< 0.1	0.1-0.9	1.0-1.4	1.5-1.8	> 1.8
Pathological casts	< 0.1	> 0.1	-	-	-
Bacteria	< 15	16-30	31-100	> 100	-
Total crystals	< 1.4	1.4-4.1	4.2-13.6	13.7-30.0	> 30.0
CaOx	< 1.4	1.4-4.1	4.2-13.6	13.7-30.0	> 30.0
Mucus	< 30	30-60	61-150	151-300	> 300
Yeasts	< 0.7	0.7-2.0	2.1-5.0	5.1-10.0	> 10.0
Sperm	< 2.7	> 2.7	-	-	-
RBC - red blood cells. WBC - white blood cells. EPI - squamous epithelial cells. NEC - non-squamous epithelial cells. CaOx - crystals - calcium oxalate.					

### Technical comparison of Atellica UAS 800 and UAS 60 analyzers

The main technical specifications and usability of Atellica UAS 800 and UAS 60, used to compare these two analyzers, are presented in Table 2. The comparison was based on assessments provided by two operators.

**Table 2:** The main technical specifications and usability of Atellica UAS 800 and UAS 60

	**Atellica UAS 800**	**Atellica UAS 60**
**Physical information**		
Depth x height x width (mm)	680 x 625 x 625	315 x 325 x 305
Weight (kg)	52	10
**System information**		
Throughput (tests/hour)	up to 106	up to 60
Storage (including images)	10,000 results	10,000 results
Waste drawer capacity	400 used cuvettes	50 used cuvettes
**Sample requirements**		
Sample aspiration	automatic	manual
Sampling volume (µL)	175	175
Required sample volume (mL)	2.0	-
**Usability**		
Ease of use	easy*	moderate*
Maintenance	manual, easy*	manual, easy*
*assessment provided by two operators.		

### Risk analysis

Through risk analysis, we assessed the potential risk to patient from omitting certain elements of urine sediment if the results from UAS 60 were not reviewed by the laboratory operator. Based on the study by Miler *et al.*, we identified 23 potential errors (E01 to E23) that represent possible missed elements in the urine sediment with negative results after automated image evaluation, assessed on a semi-quantitative scale, along with four categories of patient’s harm severity (S1 to S4) (Table 3) (*14*). Therefore, the study included samples with negative results for certain elements of urine sediment (RBC, WBC, EPI, NEC, hyaline casts, pathological casts, bacteria, total crystals, CaOx, mucus, yeasts, respectively), but positive after manual operator review. Severity levels of potential risk to the patient were assessed based on the clinical significance of the elements in the urine sediment (Table 3) (*14*).

**Table 3:** Defined errors and patient harm severity levels used for risk analysis

**Error**	**Explanation of error, missed elements (category)**
E01	Erythrocytes +1
E02	Erythrocytes +2
E03	Erythrocytes ≥ +3
E04	Leukocytes +1
E05	Leukocytes +2
E06	Leukocytes 3
E07	Squamous epithelial cells +1
E08	Squamous epithelial cells +2
E09	Squamous epithelial cells 3
E10	Non-squamous epithelial cells +1
E11	Non-squamous epithelial cells ≥ +2
E12	Hyaline casts +1
E13	Hyaline casts ≥+2
E14	Pathological casts ≥+1
E15	Bacteria +1
E16	Bacteria ≥ +2
E17	Total crystals +1
E18	Total crystals ≥ +2
E19	Crystals - calcium oxalate ≥ +1
E20	Mucus +1
E21	Mucus ≥ +2
E22	Yeasts +1
E23	Yeasts ≥ +2
**Severity level**	**Explanation of severity level of patient harm**
S1	Minimal harm to the patient, likely caused by sample contamination
S2	Requirement for a repeat sample, without causing further harm to the patient
S3	Delayed therapy resulting from missed elements in the urine sediment
S4	Misdiagnosis leading to potentially life-threatening conditions

The frequency of each error was calculated by dividing the number of samples for each error by the total number of samples included in the study (frequency (%) = number of samples for each error/463 × 100). Based on the observed frequencies of each error, five occurrence categories (O1 to O5) were defined. Occurrence category O1 refers to an error frequency of < 1.0%, O2 to 1-9%, O3 to 10-19%, O4 to 20-49% and O5 to ≥ 50%.

Risk analysis was conducted by combining the severity level of patient harm with occurrence categories in a risk acceptability matrix, as defined by ISO 14971:2019 (*15*). In this matrix, errors highlighted in green were classified as low risk, those in yellow as intermediate risk and the red area was identified as the most hazardous for patients. Errors in the red area were considered unacceptable.

### Statistical analysis

For analytical comparison, results were presented as number and percentage for each category of observed urine sediment particle and were evaluated by weighted kappa analysis. Agreement was expressed as Cohen’s kappa value. Kappa value with lower limit of 95% confidence interval ≥ 0.6 was considered acceptable. To assess agreement analysis, some categories with small number of samples/events were merged (for NEC, hyaline casts, pathological casts, total crystals, CaOx, mucus, and yeasts). Data were analyzed using MedCalc v20.008 (Ostend, Belgium) statistical software and Microsoft Office Excel 2016 (Redmond, USA).

## Results

All obtained measurements of the control samples were in complete agreement with the manufacturer’s declared values. The between-run and within-run precision for RBC were 11.3% and 10.2% for Level I (Abnormal), and both were 0.0% for Level II (Normal). For WBC, the precision values were 10.8% and 10.7% for Level I (Abnormal), and 18.1% and 17.3% for Level II (Normal). All obtained precision values were within the acceptance criteria, except for WBC at Level II (Normal), which was resulting from the low number of WBC in the control sample.

### Analytical comparison of Atellica UAS 800 and UAS 60 analyzers

Comparison of urine sediment analysis between UAS 800 and UAS 60 after the first sample pass (without operator review) on UAS 60 is presented in Table 4. An acceptable kappa value with a strong level of agreement was observed for CaOx and yeasts (kappa: 0.83 (0.69 to 0.98), and 0.94 (0.86 to1.00), respectively), while a moderate level of agreement was found for RBC, WBC and EPI (kappa: 0.75 (0.71 to 0.80), 0.78 (0.74 to 0.82), and 0.75 (0.70 to 0.80), respectively). The kappa value for bacteria and mucus indicated weak agreement, with more negative results on UAS 60 (kappa: 0.57 (0.51 to 0.62) and 0.59 (0.54 to 0.65), respectively). For NEC, weak agreement was also observed, but with more positive results on UAS 60 (kappa: 0.40 (0.31 to 0.48)). The poorest agreement was noticed for hyaline and pathological (non-hyaline) casts, with more positive results obtained on UAS 60 analyzer, and for total crystals, with more negative results on UAS 60 (kappa: 0.23 (0.17 to 0.29), 0.07 (0.00 to 0.15), and 0.36 (0.25 to 0.47), respectively). Out of the total 463 samples, only three had a positive sperm finding, but none was detected on UAS 60 by automatic image evaluation.

**Table 4:** Comparison of urine sediment analysis between UAS 800 and UAS 60 after the first sample pass (automatic image evaluation without operator review)

**Parameter**	**UAS800 (N = 463)** **N (%)**	**UAS60 (N = 463)** **N (%)**	**Weighted kappa (95% CI)**	**Agreement** **(%)**
**RBC**				
Negative	193 (42)	261 (56)		
+1	172 (37)	111 (24)		
+2	44 (9.5)	41 (8.9)	0.75 (0.71-0.80)	71.2
+3	12 (2.6)	9 (1.9)		
+4	42 (9.1)	41 (8.9)		
**WBC**				
Negative	156 (34)	205 (44)		
+1	181 (39)	133 (29)		
+2	75 (16)	78 (17)	0.78 (0.74-0.82)	74.5
+3	20 (4.3)	18 (3.9)		
+4	31 (6.7)	29 (6.3)		
**WBC - clumps**				
Negative	449 (97)	453 (98)	0.49 (0.24-0.74)	97.4
+1	14 (3)	10 (2)		
**EPI**				
Negative	272 (59)	314 (68)		
+1	106 (23)	84 (18)		
+2	62 (13)	50 (11)	0.75 (0.70-0.80)	79.5
+3	12 (2.6)	8 (1.7)		
+4	11 (2.4)	7 (1.5)		
**NEC**				
Negative	323 (70)	298 (64)		
+1	116 (25)	149 (32)	0.40 (0.31-0.48)	69.5
+2 to +4	24 (5.2)	16 (3.5)		
**Hyaline casts**				
Negative	412 (89)	215 (46)		
+1	43 (9.3)	229 (50)	0.23 (0.17-0.29)	55.1
+2 to +4	8 (1.7)	19 (4.1)		
**Pathological casts**				
Negative	458 (99)	381 (82)	0.07 (0.00-0.15)	82.9
+1	5 (1.1)	82 (18)		
**Bacteria**				
Negative	36 (7.8)	79 (17)		
+1	164 (35)	119 (26)	0.57 (0.51-0.62)	61.6
+2	177 (38)	165 (36)		
+3	86 (19)	100 (22)		
**Total crystals**				
Negative	359 (78)	443 (96)		
+1	81 (18)	8 (1.7)	0.36 (0.25-0.47)	80.1
+2 to +4	23 (5.0)	12 (2.6)		
**CaOx**				
Negative	445 (96)	450 (97)	0.83 (0.69-0.98)	98.9
+1 to +4	18 (3.9)	13 (2.8)		
**Mucus**				
Negative	199 (43)	335 (72)		
+1	164 (35)	64 (14)	0.59 (0.54-0.65)	61.8
+2	70 (15)	39 (8.4)		
+3 to +4	30 (6.5)	25 (5.4)		
**Yeasts**				
Negative	444 (96)	446 (96)	0.94 (0.86-1.00)	99.6
+1 to +4	19 (4.1)	17 (3.7)		
**Other (sperm)**				
Negative	460 (99)	463 (100)	-	99.4
+1	3 (0.6)	0 (0.0)		
CI - confidence interval. RBC - red blood cells. WBC - white blood cells. EPI - squamous epithelial cells. NEC - non-squamous epithelial cells. CaOx - crystals - calcium oxalate.				

Comparison of urine sediment analysis between UAS 800 and UAS 60 after operator review is presented in Table 5. In this case, acceptable kappa values were obtained for all analyzed parameters. For hyaline and pathological casts, after reclassification a strong level of agreement was observed, indicating false positive results by automatic image evaluation, mostly in category +1 (kappa: 0.23 (0.17 to 0.29) *vs.* 0.93 (0.90 to 0.99) and 0.07 (0.00 to 0.15) *vs*. 0.72 (0.42 to 1.00), respectively). False positive results by automatic image evaluation were also observed for NEC, after reclassification a strong level of agreement was obtained (kappa: 0.40 (0.31 to 0.48) *vs.* 0.80 (0.76 to 0.88)). On the other hand, for total crystals and mucus, automatic image evaluation resulted in false negative results. After reclassification, agreement was almost perfect (kappa: 0.36 (0.25 to 0.47) *vs.* 0.96 (0.94 to 0.99) and 0.59 (0.54 to 0.65) *vs.* 0.92 (0.89 to 0.95), respectively). False negative results by automatic image evaluation were also observed for bacteria, moderate agreement was obtained after reclassification (kappa: 0.57 (0.51 to 0.62) *vs.* 0.67 (0.62 to 0.73)). Of three samples with positive sperm findings, two were classified as positive after operator review.

**Table 5:** Comparison of urine sediment analysis between UAS 800 and UAS 60 after operator review

**Parameter**	**UAS800 (N = 463)** **N (%)**	**UAS60 (N = 463)** **N (%)**	**Weighted kappa (95% CI)**	**Agreement** **(%)**
**RBC**				
Negative	193 (42)	242 (52)		
+1	172 (37)	133 (29)	0.78 (0.74-0.82)	74.9
+2	44 (9.5)	37 (8.0)		
+3	12 (2.6)	12 (2.6)		
+4	42 (9.1)	39 (8.4)		
**WBC**				
Negative	156 (34)	176 (38)		
+1	181 (39)	170 (37)		
+2	75 (16)	70 (15)	0.84 (0.81-0.87)	81.6
+3	20 (4.3)	18 (3.9)		
+4	31 (6.7)	29 (6.3)		
**WBC - clumps**				
Negative	449 (97)	447 (97)	0.58 (0.39-0.73)	98.1
+1	14 (3)	16 (3.5)		
**EPI**				
Negative	272 (59)	278 (60)		
+1	106 (23)	125 (27)		
+2	62 (13)	42 (9.1)	0.77 (0.72-0.82)	81.6
+3	12 (2.6)	10 (2.2)		
+4	11 (2.4)	8 (1.7)		
**NEC**				
Negative	323 (70)	357 (77)		
+1	116 (25)	88 (19)	0.80 (0.75-0.88)	98.7
+2 to +4	24 (5.2)	18 (3.9)		
**Hyaline casts**				
Negative	412 (89)	413 (89)		
+1	43 (9.3)	38 (8.2)	0.93 (0.90-0.99)	98.9
+2 to +4	8 (1.7)	12 (2.6)		
**Pathological casts**				
Negative	458 (99)	457 (99)	0.72 (0.42-1.00)	99.4
+1	5 (1.1)	6 (1.3)		
**Bacteria**				
Negative	36 (7.8)	34 (7.3)		
+1	164 (35)	176 (38)	0.67 (0.62-0.73)	72.8
+2	177 (38)	165 (36)		
+3	86 (19)	88 (19)		
**Total crystals**				
Negative	359 (78)	356 (77)	0.96 (0.94-0.99)	98.9
+1	81 (18)	87 (19)		
+2 to +4	23 (5.0)	20 (4.3)		
**CaOx**				
Negative	445 (96)	442 (96)	0.95 (0.89-1.00)	99.8
+1 to +4	18 (3.9)	21 (4.5)		
**Mucus**				
Negative	199 (43)	197 (43)		
+1	164 (35)	197 (43)	0.92 (0.89-0.95)	92.1
+2	70 (15)	46 (9.9)		
+3 to +4	30 (6.5)	23 (5.0)		
**Yeasts**				
Negative	444 (96)	444 (96)	1.00 (1.00-1.00)	100.0
+1 to +4	19 (4.1)	19 (4.3)		
**Other (sperm)**				
Negative	460 (99)	461 (99)	0.80 (0.41-1.00)	99.8
+1	3 (0.6)	2 (0.4)		
CI - confidence interval. RBC - red blood cells. WBC - white blood cells. EPI - squamous epithelial cells. NEC - non-squamous epithelial cells. CaOx - crystals - calcium oxalate.				

### Technical comparison of Atellica UAS 800 and UAS 60 analyzers

Comparison of technical performance between Atellica UAS 800 and UAS 60 analyzers is presented in Table 2. In terms of physical dimensions, UAS 800 is significantly larger and heavier, compared to the more compact UAS 60. Furthermore, UAS 800 has a higher throughput and waste drawer capacity, while data storage capacities are the same for both analyzers. In terms of sample requirements, UAS 800 performs automatic sample aspiration, while UAS 60 requires manual aspiration. Both analyzers use the same sampling volume, though UAS 800 requires a minimum sample volume. Regarding usability, UAS 60 is considered more demanding compared to UAS 800. Both analyzers require the same maintenance procedure, which is considered easy to perform.

### Risk analysis

Of possible 23, we identified 15 errors. Corresponding severity level of potential risk to patient, frequency and occurrence category of each error are presented in Table 6. The most frequent errors were unrecognized total crystals and mucus in category +1 (O4 = 30.0 and O3 = 17.1%, respectively) with severity level S1 and S2, respectively. No errors were found for RBC, WBC, EPI, NEC and hyaline casts in categories ≥ +2.

**Table 6:** Severity level of potential risk to patient, frequency and occurrence category of each error (negative results after automated image evaluation on UAS 60, but positive after operator review)

**Error**	**Missed elements (category)**	**Severity level**	**N**	**Frequency (%)**	**Occurrence category**
E01	Erythrocytes +1	S1	23	5.0	O2
E02	Erythrocytes +2	S3	0	0.0	O1
E03	Erythrocytes ≥ +3	S4	0	0.0	O1
E04	Leukocytes +1	S1	32	6.9	O2
E05	Leukocytes +2	S2	0	0.0	O1
E06	Leukocytes ≥ +3	S4	0	0.0	O1
E07	Squamous epithelial cells +1	S1	40	8.6	O2
E08	Squamous epithelial cells +2	S3	0	0.0	O1
E09	Squamous epithelial cells ≥ +3	S4	0	0.0	O1
E10	Non-squamous epithelial cells +1	S2	2	0.4	O1
E11	Non-squamous epithelial cells ≥ +2	S3	0	0.0	O1
E12	Hyaline casts +1	S1	1	0.2	O1
E13	Hyaline casts ≥ +2	S3	0	0.0	O1
E14	Pathological casts ≥ +1	S4	1	0.2	O1
E15	Bacteria +1	S1	44	9.5	O3
E16	Bacteria ≥ +2	S2	2	0.4	O1
E17	Total crystals +1	S2	79	17.1	O3
E18	Total crystals ≥ +2	S3	11	2.4	O1
E19	Crystals - calcium oxalate ≥ +1	S2	10	2.2	O1
E20	Mucus +1	S1	139	30.0	O4
E21	Mucus ≥ +2	S2	1	0.2	O1
E22	Yeasts +1	S2	4	0.9	O1
E23	Yeasts ≥ +2	S3	3	0.6	O1

The risk acceptability matrix, which combines patient harm severity with occurrence categories of errors, is presented in Table 7. All identified errors were classified as low risk, except for three errors that were categorized as intermediate risk. Among the intermediate risk errors, one was associated with severity level S4 (missing to report pathological casts ≥ +1) with a frequency < 1.0% (N = 1, O1 = 0.2%). Another was linked to severity level S2 (missing to report total crystal +1, O3 =17.1%) and the third was a combination of the highest occurrence level (O4) with the lowest severity level S1 (missing to report mucus +1, O4 = 30.0%). No errors were found in the red area.

**Table 7:** The risk acceptability matrix with combination of patient harm severity level and occurrence categories of errors

	**S1**	**S2**	**S3**	**S4**
**O5**				
**O4**	E20			
**O3**	E15	E17		
**O2**	E01, E04, E07			
**O1**	E12	E10, E16, E19, E21, E22	E18, E23	E14
Errors highlighted in green were classified as low risk, those in yellow as intermediate risk and the red area was identified as the most hazardous for patients. Errors in the red area were considered unacceptable. E - error. S - severity of patient harm. O - occurrence category of error.				

Through risk analysis, we assessed the potential patient risk from omitting certain elements of urine sediment. However, for NEC, hyaline casts and pathological casts, a high frequency of false positive results after automated image evaluation on UAS 60 was observed, rather than instances of omission (Table 8). The highest frequency of false positive results was noted for hyaline casts in category +1 (N = 196, 42.3%).

**Table 8:** Frequency of false positive results after automated image evaluation on UAS 60 analyzer (negative results after operator review)

**Urine sediment parameter (category)**	**N (false positive)**	**Frequency (%)**
Non-squamous epithelial cells +1	61	13.2
Non-squamous epithelial cells ≥ +2	0	0.0
Hyaline casts +1	196	42.3
Hyaline casts ≥ +2	3	0.6
Pathological casts ≥ +1	77	16.6

## Discussion

In terms of analytical specifications, we reported that automatic image identification on UAS 60 resulted in false negative results for bacteria, total crystals, and mucus, indicating reduced sensitivity for these urine sediment particles. For bacteria, this finding was somewhat surprising, given that most studies have reported false positive rates with digital image-based systems (*11*, *16*). It is important to note that the majority of these false negative results, after operator review, were classified in the +1 category, which is a result that usually does not indicate a urinary tract infection, but rather improper urine collection or prolonged storage and transportation at temperatures higher than recommended (*14*). Nikolac *et al.* highlighted that patients are often not fully informed about all preanalytical requirements, such as using the appropriate container, delivering the sample properly to the laboratory, and performing proper genital cleaning before urine collection (*17*). Failure to adhere to these requirements can compromise the accuracy of the test results, emphasizing the need for better patient education in the preanalytical phase of laboratory testing. Although some studies have shown satisfactory sensitivity and specificity for bacteria detection by automated urine sediment analyzers, urine culture is still the gold standard for detecting bacteria in urine and diagnosing urinary tract infections (*16*, *18*). After operator review, the majority of false-negative results for total crystals and mucus were also classified in the +1 category, which typically does not indicate pathological conditions. Urinary crystals usually suggest supersaturation with certain substances and can result from genetic disorders, metabolic issues, or medications. However, their presence does not always signal a metabolic or renal problem, as they can also occur under normal physiological conditions, especially at low concentrations (*19*). A normal urine test usually shows a small to moderate amount of mucus. An increased quantity of mucus might indicate conditions such as urinary tract infection, sexually transmitted disease, kidney stones, irritable bowel syndrome, or bladder cancer (*20*). It is interesting to note that, when comparing results before and after operator review from the UAS 60, false negative results were observed after automatic image evaluation for RBC and WBC, indicating somewhat reduced sensitivity for these urine sediment particles. These are very important categories in urine sediment analysis, as a high number of RBC and WBC can indicate kidney diseases, infection, or malignant conditions (*14*). However, these false negative results occurred in 22 samples for RBC (4.8%) and 29 samples for WBC (6.3%). After operator review, all of these samples were classified into category +1, which does not suggest the presence of severe hematuria or infection. Furthermore, although the kappa value was acceptable, it is noteworthy that differences were observed in the number of negative results for RBC, WBC and NEC between UAS 800 and UAS 60 after operator review. More negative results were obtained on UAS 60. We believe the main reason for this is the time delay in analysis. Samples were first analyzed on the UAS 800 and then on the UAS 60. Although the maximum time between analysis on the UAS 800 and the UAS 60 was one hour, some particles may have degraded due to their fragility before being analyzed on the UAS 60. Therefore, the observed false negative results obtained through automatic image identification on UAS 60 have no significant clinical impact on the clinical decision-making process.

On the other hand, automatic identification on the UAS 60 resulted in false positive results for NEC, hyaline, and pathological casts. Large leukocytes and EPI were mostly classified as NEC, while numerous particles, such as crystal clusters, mucus with cells or crystal clusters, *etc.*, were classified as hyaline and pathological casts. Higher false positive rates for casts with digital image-based system, compared to flow cytometry-based systems, have also been reported in several previous studies (*11*, *21*). Cho *et al*. observed that tendency to produce false positive results is greater in the presence of mucus, fibers and other contaminants in urine sample (*11*). The presence of NEC indicates tubular damage from various nephron regions, and a high number may be linked to acute kidney injury (*22*). While a small number of hyaline casts can be found in the urine of healthy individuals, a higher number of these casts, along with other types of casts (*e.g.* cellular, granular, *etc.*) might indicate glomerular or tubular conditions, such as glomerulonephritis or pyelonephritis, or exposure to nephrotoxic substances (*23*).

After operator review and reclassification of misclassified urine sediment particles, obtained results were comparable to UAS 800 for all analyzed parameters. This should confirm that despite the constant improvement of automated urine sediment analyzers, review by experienced laboratory personnel is still necessary to accurately confirm classified urine sediment particles. However, we conducted a risk analysis of potential patient harm if results from the UAS 60 were not reviewed by laboratory personnel. The results indicated a low risk of patient harm for almost all defined errors, except for three errors classified as intermediate risk: unrecognized mucus and total crystals in category +1 and unrecognized pathological casts. As previously mentioned, mucus and total crystals at low concentrations are typically found under normal physiological conditions, so their unrecognition in category +1 should not place patients at risk of diagnostic errors. The presence of unrecognized pathological casts was associated with the highest severity level (S4) due to their significance in the diagnosis of renal diseases. Although this error was categorized as intermediate risk, it is important to note that it was identified in only one sample (0.2%). As previously stated, we observed false positive results after automatic image evaluation on UAS 60 for NEC, hyaline and pathological casts. The highest frequency of false positives was noted for hyaline casts. While these errors do not present a direct potential harm to patients, they can lead to unnecessary repeated sampling and/or additional analyses. Therefore, every positive result for NEC, hyaline and pathological casts after automatic image evaluation should be reviewed by laboratory personnel. This comparison of results from the UAS 60 before operator review provides significant information, indicating that UAS 60 analyzer produces appropriate results for clinicians without the risk of diagnostic errors. Therefore, in emergency situations where laboratory analysis is not available, it could serve as an effective urine sediment analyzer or potentially as a point-of-care analyzer in the future.

Comparing the analyzers main technical characteristics, it is visible that UAS 60 is a smaller and lighter analyzer, which can be advantageous for laboratories with limited space. However, throughput and waste drawer capacity of the UAS 60 is significantly lower, which can limit its use in laboratories with a high volume of samples. Contrary to UAS 800 where sample aspiration is automatic, UAS 60 requires manual sample pipetting into cuvettes. In terms of time-consumption, this can be a disadvantage and a major limiting factor for routine use in large laboratories. However, regarding carry-over, manual pipetting can reduce risk of false results. Due to automatic sample aspiration, the UAS 800 requires a minimum sample volume of 2.0 mL, which can be challenging for pediatric population and neonates. Therefore, the UAS 60 may be more suitable for analyzing these types of samples. However, it is important to note that, according to study by Bunjevac *et al.*, smaller volume of urine can lead to false negative results, particularly for RBC, WBC, EPI and pathological casts (*24*). This can result in serious mistakes in diagnosis, monitoring, management and treatment of renal patients. They recommended that urine sample volume should be no less than 10 mL to ensure accurate results.

In this study, we compared only categorical results, lack of numerical data comparison may be a limitation. Additionally, for EPI, pathological casts and sperm we were unable to collect 10 samples *per* category (despite merged categories) which led to underestimated kappa values but a high percentage of agreement. Furthermore, unreviewed results from the UAS 800 analyzer were not collected. Nevertheless, this study provides valuable insights, results from the Atellica UAS 60 are evaluated for the first time and obtained findings could be highly useful to clinicians and laboratory professionals.

In conclusion, Atellica UAS 60 is a fast, lightweight, and user-friendly automated urine sediment analyzer that provides results comparable to UAS 800. The quality of reported results without operator review is not compromised, as clinicians have access to nearly the same clinical information and patients are not at risk of diagnostic errors. However, positive results for NEC, hyaline and pathological cast should still be reviewed. Atellica UAS 60 is suitable for automating urine sediment analysis in low- to mid-volume laboratories and can serve as an effective backup instrument for Atellica UAS 800 in larger laboratories.

## Data Availability

The data generated and analyzed in the presented study are available from the corresponding author on request.
